# A103 A COMPREHENSIVE REVIEW OF ENTEROLITH ILEUS SECONDARY TO COMPLICATIONS OF ILEAL DIVERTICULA AND INVESTIGATIONS INTO OPTIMAL MANAGEMENT

**DOI:** 10.1093/jcag/gwad061.103

**Published:** 2024-02-14

**Authors:** C J Miranda, A Aijaz, A M Carlson, N Hossein-Javaheri, M H Ali, M Ismail

**Affiliations:** Gastroenterology, University at Buffalo, Buffalo, NY; Gastroenterology, University at Buffalo, Buffalo, NY; Gastroenterology, University at Buffalo, Buffalo, NY; Internal Medicine, University at Buffalo, Buffalo, NY; Gastroenterology, University at Buffalo, Buffalo, NY; Internal Medicine, University at Buffalo, Buffalo, NY

## Abstract

**Background:**

Acquired ileal diverticula are rarely found in adults and are only apparent in 1.9% of small bowel contrast studies. Even more rarely, these diverticula can lead to enterolith formation which can obstruct the bowel, a term coined “enterolith ileus”. Both surgical and non-surgical approaches have been espoused in tackling this complication but definitive literature on optimal management is lacking and is limited to scattered case reports and case series. With this phenomenon carrying significant morbidity and mortality, we have conducted a comprehensive review of all available case reports to summarize our understanding of enterolith ileus secondary to ileal diverticula.

**Aims:**

To understand the varying clinical presentations of enterolith ileus from ileal diverticula complications and understanding optimal management for the pathology.

**Methods:**

A comprehensive search of PubMed, OVID, CINAHL, and Cochrane databases up to February 2023 was conducted to identify all studies reporting clinical information on enterolith formation in ileal diverticula leading to small bowel obstruction. Relevant data were extracted from selected studies to determine clinical courses and optimal management.

**Results:**

Our literature review identified 61 case reports of enterolith ileus secondary to complications of ileal diverticula. The mean age of the patients was 51.64 years (SD 20.9), with 27% of them being female. The most common symptoms reported were abdominal pain (87%), nausea, and vomiting (51%), followed by constipation (18%) and melena (5%). Notably, localized abdominal tenderness was a common finding, observed in 74% of cases across 29 out of 34 studies reporting physical examination findings. Diagnostic investigations, including abdominal X-rays and CT scans, consistently revealed dilated bowel loops and signs of bowel obstruction. Surgical intervention was eventually required for almost all patients (97%), with diverticulectomy performed in 47%, enterectomy in 12%, and surgical resection of the bowel in 32% of cases respectively. These surgical interventions demonstrated high success rates, effectively resolving the condition in most cases. Complications, such as wound infection (2 cases), postoperative ileus (3 cases), anastomotic leak, and wound dehiscence (1 case each) were observed in a limited number of patients.

**Conclusions:**

Our findings highlight the critical importance of prompt diagnosis and management of enterolith ileus secondary to complications of ileal diverticula. We recommend avoidance of initial conservative management as surgical intervention has proven almost always necessary and is highly successful in tackling this pathology. Early recognition and intervention can help minimize the risk of morbidity and mortality associated with this condition.

**Funding Agencies:**

None

